# Role of Early Life Cytotoxic T Lymphocyte and Natural Killer Cell Immunity in Paediatric HIV Cure/Remission in the Anti-Retroviral Therapy Era

**DOI:** 10.3389/fimmu.2022.886562

**Published:** 2022-05-11

**Authors:** Vinicius A. Vieira, Nicholas Herbert, Gabriela Cromhout, Emily Adland, Philip Goulder

**Affiliations:** ^1^ Peter Medawar Building for Pathogen Research, Department of Paediatrics, University of Oxford, Oxford, United Kingdom; ^2^ Africa Health Research Institute (AHRI), Nelson R Mandela School of Medicine, Durban, South Africa; ^3^ HIV Pathogenesis Programme, Doris Duke Medical Research Institute, Nelson R Mandela School of Medicine, University of KwaZulu-Natal, Durban, South Africa

**Keywords:** HIV, innate immunity, CTL, NK cell, paediatric

## Abstract

Only three well-characterised cases of functional cure have been described in paediatric HIV infection over the past decade. This underlines the fact that early initiation of combination antiretroviral therapy (cART), whilst minimising the size of the viral reservoir, is insufficient to achieve cure, unless other factors contribute. In this review, we consider these additional factors that may facilitate functional cure in paediatric infection. Among the early life immune activity, these include HIV-specific cytotoxic T-lymphocyte (CTL) and natural killer (NK) cell responses. The former have less potent antiviral efficacy in paediatric compared with adult infection, and indeed, in early life, NK responses have greater impact in suppressing viral replication than CTL. This fact may contribute to a greater potential for functional cure to be achieved in paediatric versus adult infection, since post-treatment control in adults is associated less with highly potent CTL activity, and more with effective antiviral NK cell responses. Nonetheless, antiviral CTL responses can play an increasingly effective role through childhood, especially in individuals expressing then ‘protective’ HLA-I molecules HLA-B*27/57/58:01/8101. The role of the innate system on preventing infection, in shaping the particular viruses transmitted, and influencing outcome is discussed. The susceptibility of female fetuses to *in utero* mother-to-child transmission, especially in the setting of recent maternal infection, is a curiosity that also provides clues to mechanisms by which cure may be achieved, since initial findings are that viral rebound is less frequent among males who interrupt cART. The potential of broadly neutralising antibody therapy to facilitate cure in children who have received early cART is discussed. Finally, we draw attention to the impact of the changing face of the paediatric HIV epidemic on cure potential. The effect of cART is not limited to preventing AIDS and reducing the risk of transmission. cART also affects which mothers transmit. No longer are mothers who transmit those who carry genes associated with poor immune control of HIV. In the cART era, a high proportion (>70% in our South African study) of transmitting mothers are those who seroconvert in pregnancy or who for social reasons are diagnosed late in pregnancy. As a result, now, genes associated with poor immune control of HIV are not enriched in mothers who transmit HIV to their child. These changes will likely influence the effectiveness of HLA-associated immune responses and therefore cure potential among children.

## Introduction

Four decades have passed since the first case report of HIV/AIDS in 1981 (1), marking the onset of a pandemic that has devastated entire populations and killed more than 32 million people globally (2). Although, theoretically, we now have the tools *via* combination antiretroviral therapy (cART) to end the HIV/AIDS pandemic and block adult-to-adult and mother-to-child transmission, an estimated 1.5m new HIV infections occurred in 2020, of which approximately 10% or 150,000 were children (3).

With advances in early infant diagnosis and improved access to cART in children over the past decade there has been an increase in the number of children living with HIV (CLHIV) who are accessing cART, from 417,000 in 2010 to 924,000 in 2020. Of those CLHIV who are accessing cART, only approximately 40% have achieved viral suppression in 2020 (2, 3). Children are particularly affected by the challenges that lifelong cART treatment started from birth presents, including those of cART adherence, toxicity and the uncertain impact on normal childhood development (4). This prompts the importance of developing new strategies to optimise treatment (e.g. long-acting antiretrovirals) and/or novel therapeutic tools that can lead to cure/remission in children.

To date, only three adults have been considered truly cured of HIV, in whom no replication competent virus was found after treatment interruption (5–7). Both patients underwent bone-marrow transplant from a donor with a homozygous CCR5 delta-32 mutation after chemotherapy due to associated haematological malignancy. Albeit an undeniable accomplishment, the complex road to achieving cure in these two cases is not a viable strategy for people living with HIV who do not have otherwise untreatable malignancies (8). Meanwhile, reports of viral remission (‘functional cure’) solely achieved by the early start of antiretroviral therapy (ART) both in adults (9–11) and children (12–14) were received with great enthusiasm by the scientific community. Remission means undetectable plasma HIV-RNA by the standard assays in the absence of cART, while HIV-DNA remains at low levels in latently infected cells. However, clinical studies aiming to diagnose HIV perinatal transmission soon after birth and start therapy at a very early stage of the disease course failed to replicate these cases of functional cure in children (15–17).

It is now known, with rare exceptions, that remission will not be primarily achieved by cART alone. Nonetheless, early diagnosed and virologically suppressed children will have minimal size and diversity of viral reservoir by the time other immune interventions can be implemented, and advantage can be taken of a more mature immune system. The ideal age to implement a new therapy and stop ART is uncertain, but optimally it should happen before late childhood and adolescence when the struggle with adherence hits hardest. Interestingly, this exact period between 4 and 12 years is called the “honeymoon period of infectious diseases” by immunologists (18), during which outcome from many infections is superior to that observed in younger and older age groups. Although equally susceptible to infection, children at this stage have lower mortality and morbidity from several other infections, including tuberculosis, influenza, mumps, measles, and Varicella-Zoster (18, 19). Partly for this reason, it has not been surprising that children have, with rare exceptions, suffered little in the way of severe disease during the SARS-CoV-2 pandemic (20–22). Although not yet completely elucidated, this period of life may represent the perfect balance between developing an immune response and the harmful damage it may provoke. The innate response and the priming of the adaptive response are likely both more efficient and better regulated at this age than in adults. Hence, recovery from most infections, even HIV (23) may be optimal at this time, while the risk of immunopathology and autoimmune disease remains low.

In this review, we discuss the unique features of the host-pathogen interaction in the context of mother-to-child transmission (MTCT) of HIV and their impact on the potential to achieve HIV cure/remission in infected children.

## Higher HIV Cure Potential in Children Compared With Adults

**Figure 1:**
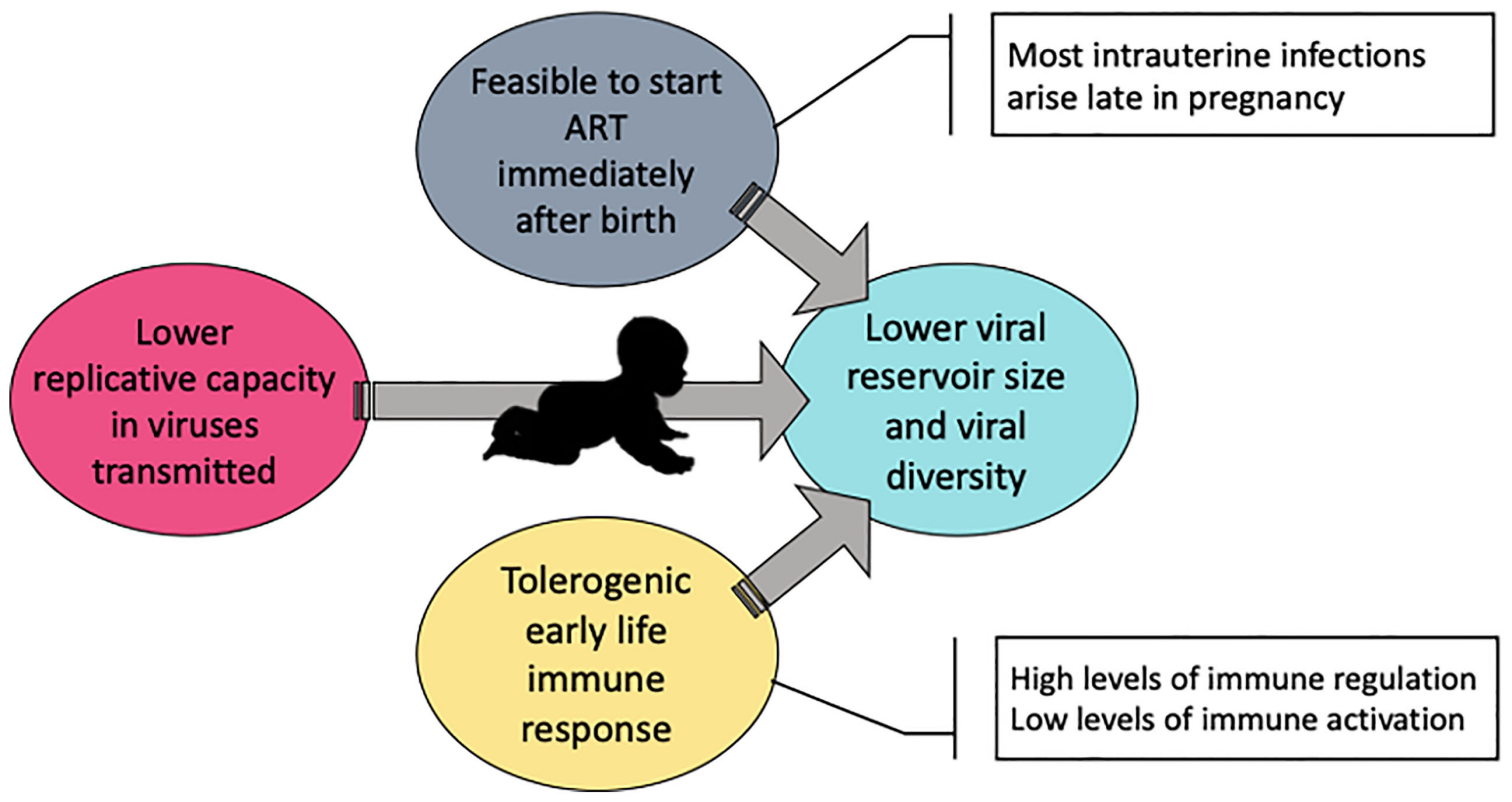
Key factors contributing to increased HIV cure potential in children with HIV.

It has been proposed that there is a greater likelihood of achieving HIV cure in children than in adults, for three principal reasons (**Figure 1**). The first of these is that cART can usually be initiated early in the course of paediatric, but not adult infection. In the absence of any intervention, the risk of vertical transmission can reach 40% (5–10% intrauterine, 15–20% intrapartum and 10–20% *via* breastfeeding) (24, 25). Substantial reductions in MTCT rates have been achieved *via* cART administered to mothers with HIV and by ART prophylaxis given to HIV-exposed infants immediately after birth. Nonetheless, intrauterine and post-partum transmission remain an issue because of cART non-adherence and maternal seroconversion during pregnancy (15) and post-partum during breast-feeding. Early cART initiation, especially following intrauterine transmission, which usually arises in the final weeks of pregnancy (26), is therefore more readily achievable in paediatric infection compared to adults, when HIV infection is usually only diagnosed after months or years of transmission. Early suppressed infants have lower total (27–29) and intact proviral (16) HIV-DNA and lower polyclonal expansion (16) and diversity (30, 31) of their viral reservoir compared with adults.

In the context of early cART, the phenotype of the inherited virus in vertical transmission in combination with the unique features of the immune system in infants impose complementary barriers to viral seeding, placing children at a theoretical advantage over adults to achieve remission (**Figure 1**). The replicative capacity of the virus transmitted to the child is on average lower than the circulating viruses found in the mother (32, 33). The reasons for this are unknown but if the variants that improve transmission also typically reduce replicative capacity this would be the result. Conversely, in horizontal transmission, the transmitted founder virus is usually of high fitness, capable of rapidly establishing the infection (34). Lower viral replicative capacity (VRC) in the transmitted virus has been associated with higher CD4+ T-cell count and slower disease progression in the recipient following horizontal and vertical transmissions (32, 35). Infections established by viruses with lower VRC induce less proinflammatory cytokines during acute infection in adults and lower CD4+ and CD8+ T-cell immune activation and exhaustion (36). Moreover, high VRC is associated with higher viral reservoirs, including in the long-lived subset of naïve and central memory CD4 T-cells (36). Therefore, the low VRC of the transmitted virus in MTCT synergises with early cART in curbing the establishment of a large viral reservoir while preventing systemic inflammation and T-cell exhaustion.

In addition to these factors, the tolerogenic immune system found in infants can also be of advantage in the context of early suppressed children. In this review we use the term “tolerogenic” to summarise in a word the functionally distinct nature of early life immunity that is the subject of other reviews (37–41). The apparently immature infants’ immune response in fact is a well-adapted system to face the obstacles of the intense intrauterine maternal-fetal interplay and the immediate post-birth environment of an almost naïve immune system exposed to potentially life-threatening extracellular pathogens. The adaptations of the early life immune response are, however, exploited by intracellular pathogens as HIV, and children progress faster than adults to AIDS in the absence of ART (37). Antigen presenting cells in infants are efficient in their function, but they secrete cytokines that prime the differentiation of naive CD4+ T-cells to regulatory T-cells, Th2, Th17 and Tfh CD4+ T-cells, *via* cytokines such as Interleukin (IL)-10, IL-6, IL-23, and IL-21, to the detriment of a Th1 response that requires secretion of IL-12 and IFN-γ (38). The immune strategy adopted in early life avoids the harmful effect of a Th1 response and protects infants against life-threatening extracellular bacteria and fungi (39) but makes them more susceptible to HIV. HIV-specific CD8+ T-cells are fundamental in controlling viraemia (40, 41) and the cytokines secreted by Th1 CD4+ T-cell play a fundamental role in their priming and differentiation (42, 43). Although detrimental in the natural course of HIV infection, the tolerogenic environment in early life can be exploited in the context of early initiation of ART by maintaining very low levels of T-cell immune activation and CCR5 expression, adding another barrier to viral seeding while preserving the effector potential of these T-cells. Indeed, early treated infants have lower levels of immune activation in both CD4+ and CD8+ T-cells and have a more polyfunctional HIV-specific response when compared to those with delayed ART (16).

Overall, the possibility to initiate ART immediately after birth in all *in utero*-infected children born to mothers with HIV, the lower replicative capacity of the virus transmitted from mother-to-child and the tolerogenic immune response in early life, with high levels of immune regulation and low activation, impose barriers to viral seeding and, after years of viral ART-suppression, the size and diversity of the viral reservoir achieved can be minimal. This provides a promising starting point for immunotherapeutic interventions to operate in conjunction with key antiviral immune responses to bring about cure/remission in children.

## Antiviral Immune Responses Important for Remission: Cytotoxic T-Lymphocyte Activity

Infants are at an apparent disadvantage over adults considering that a more pro-inflammatory and aggressive HIV-specific CD8+ T-cell and Th1 responses are more effective in suppressing viraemia (44), opposing the tolerogenic and Th2 biased responses observed in children. The emergence of HIV-specific T-cell immunity is temporally associated with reaching spontaneous viraemic control in adults (40, 41). The breadth and magnitude of HIV-specific CD8+ T-cell responses, particularly those targeting Gag, have been associated with lower viral setpoint and slower progression in adults (45, 46). The two most extensive genome-wide association studies looking at single-nucleotide polymorphisms consistently identified the HLA-I genes as the key regions associated with elite control (47, 48). Elite controllers (ECs) are enriched with particular HLA-B molecules, such as HLA-B*27, HLA-B*57, HLA-B*58:01 and HLA-B*81:01 (46, 49–51). As a shared feature, these HLA-I molecules can bind and present peptides from conserved, immunogenic regions of the virus, such as Gag and Pol, escape from which therefore typically comes at a cost to viral replicative capacity (52–54). ECs are enriched with Gag-specific CD8+ T-cells, while non-controllers frequently target regions with a lower fitness barrier, such as Env and Nef (46, 55–57). Contrastingly, HIV-specific CD8+ T-cells detected in infants usually target non-Gag regions, have lower magnitude and are less polyfunctional (58–61). Moreover, HIV-specific CTLs encounter additional obstacles in vertical transmission due to infants inheriting a virus that can be already pre-adapted to half of their HLA-I alleles and, therefore, unable to target the transmitted virus effectively (62).

As children develop, the immune system matures to become more adult-like in the responses made. HIV-specific CD8+ T-cell responses increase in magnitude and breadth with age and a viral set point is achieved by the age of 5 years (63–66). Although neither breadth nor magnitude of the HIV-specific CD8+ T-cell response differentiates viraemic non-controllers and viraemic controllers, the latter markedly target Gag and Pol proteins while the former mostly target Env, Nef and non-structural proteins with low fitness cost to the virus (61). The increased naïve repertoire in children contributes to their superior ability to develop novel, variant-specific responses compared to adults (61, 67) and, by the time their T-cell response is capable of exerting selection pressure on the virus, they can lead the virus to a progressive evolutionary *cul*-*de-sac* where the already crippled virus has no further scope to escape without even greater cost to replicative capacity. The success of this ‘cornering’ strategy in maintaining disease non-progression may depend critically on the efficacy of the variant-specific CTL response (36, 64, 68). It has been previously modelled as a possible vaccine strategy that can be better exploited in children than adults (69, 70).

Viraemic control is rare in the paediatric population, and paediatric ECs (PECs) are at least tenfold less prevalent than adult ECs (71). Elite control in adults is strongly linked with HIV-specific CTLs, in special those targeting epitopes loaded by HLA-I alleles such as HLA-B*27, HLA-B *57, HLA-B *58:01 and HLA-B *81:01 (49–51). These alleles do not have the same effect in children, and indeed they are only weakly (if at all) associated with better viral control in paediatric infection (32, 72). Nonetheless, HLA-I alleles only explain 20% of viraemic control in adults (48) and other factors play an active role in adult viraemic control, including polyfunctionality and clonal expansion capacity of the HIV-specific CTL response (73–78). Indeed, we recently showed that PECs have a stronger polyfunctional profile of Gag-specific CD8+ T-cell response than viraemic slow progressors in the absence of the HLA-I alleles that are protective in adult infection (79). Like PECs, individuals who achieved remission after ART interruption, also known as post-treatment controllers (PTCs), are not enriched with the ‘protective’ alleles overexpressed in adult ECs (10). Moreover, both PECs and PTCs have lower levels of T-cell immune activation during the period of viraemic control (10, 79). Viraemic setpoint in adults is usually achieved within the first 6 weeks (80) or certainly year of infection (81), and the level of T-cell activation is directly correlated with viral setpoint (44), reflecting the priming and expansion of HIV-specific T-cell effector subsets. Contrastingly, children usually take 5 or years to achieve viral setpoint or elite control, and a tolerogenic immune environment that supports viral replication without disease progression anticipates viral control in PECs (71, 79).

Far more common than PECs, nearly 10% of ART-naïve children reach late childhood with normal-for-age CD4+ T-cell count and no sign of clinical disease, referred to as paediatric slow progressors or paediatric non-progressors (PNP) (82, 83). Although viraemic, the immune strategy adopted by PNPs can offer important insights for remission strategies in children on cART. PNPs share profound similarities to the natural hosts of simian immunodeficiency virus (SIV) infection (66, 84, 85). These African non-human primates have coevolved with SIV towards a non-aggressive and indulgent state allowing viral replication but without the systemic inflammation and progression to AIDS that typically accompanies persistent high viraemia in HIV-infected human adults. Like the natural hosts of SIV, PNPs have lower levels of systemic inflammation and T-cell activation and exhaustion compared to paediatric progressors, despite similar viraemia (66). CCR5 expression on CD4+ T-cells is lower in the central memory subset of PNPs, confining viral replication to the anyway short-lived population of effector memory cells (66). The more tolerogenic immune system observed in early life may explain the higher frequency of viraemic slow-progressors in the paediatric population. By contrast, adult viraemic non-progressors are exceedingly rare, with <50 individuals described in the literature (for example, Choudary et al. (86), 3 individuals described; Rotger et al (84), n=6; Klatt et a.l (87), n=9) and far-out-numbered by adult ECs. Early viral suppression of *in utero*-infected infants offers a unique opportunity to prevent irreversible immune activation in the tolerogenic setting of early life and confine the viral reservoir to short lived subsets until improved effector functions can be induced later in childhood.

**Figure 2:**
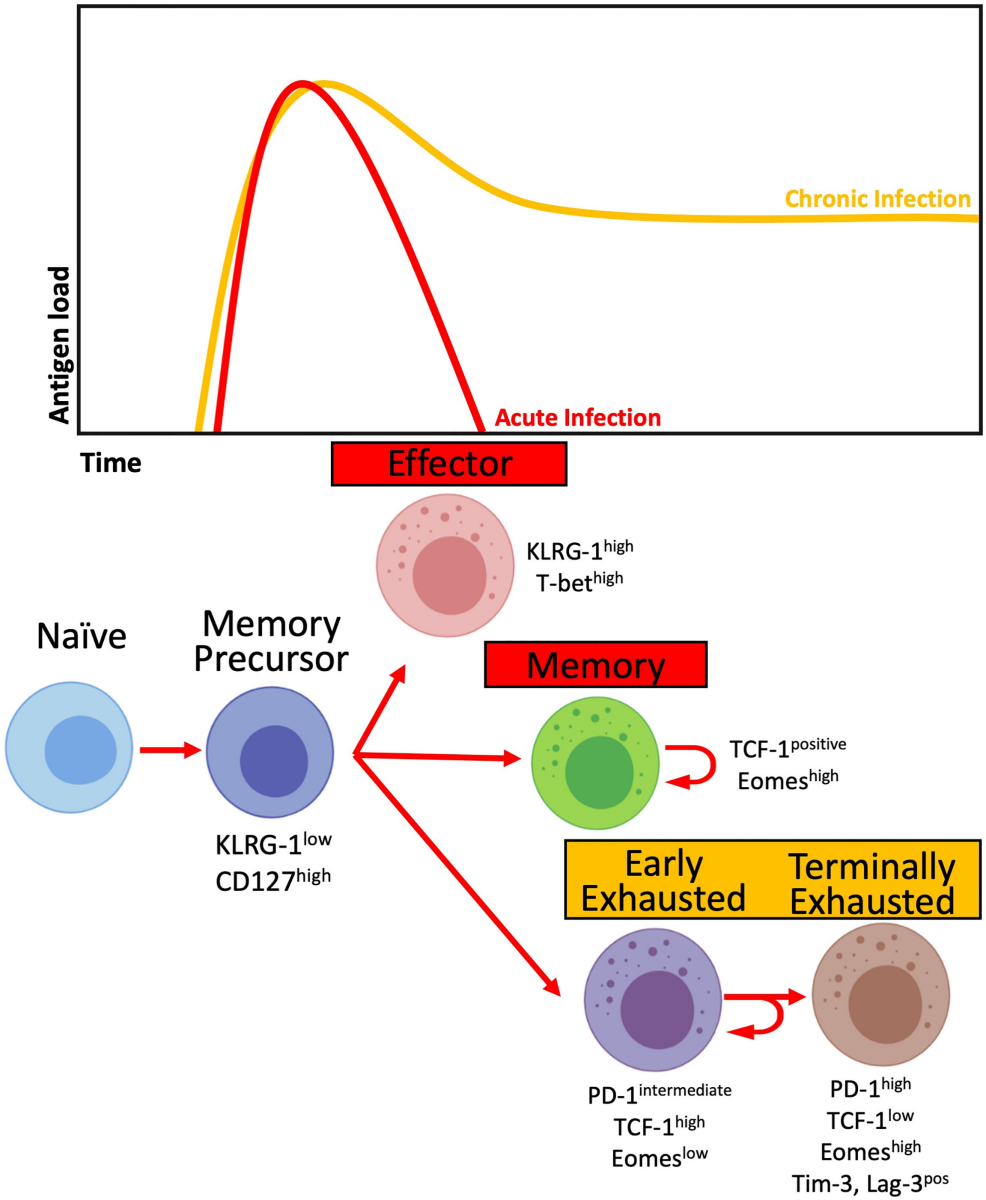
T-cell differentiation in the context of acute (red) and chronic (yellow) infections. Activated T-cells differentiate into Memory precursors cells, giving rise to Effector cells capable of controlling antigen load. In the contraction phase, most Effector cells die, while a proportion will differentiate into Memory cells. In the context of chronic antigen load, Memory precursor cells differentiate into Early Exhausted T-cells with intermediate levels of PD-1 expression and high expression of TCF-1 while retaining their capacity for self-renewal. The chronic presence of antigen leads to the differentiation of Early Exhausted cells into Terminally Exhausted with progressively decreased effector and self-renewal capacity while increasing the expression of inhibitory receptors, including PD-1, Tim-3 and Lag-3.

T-cell immune activation (HLA-DR and CD38 expression) and exhaustion (PD-1 expression) have been constantly associated with poor outcomes and HIV disease progression in adults that are only partially reversed with cART (88–90). Moreover, they predict faster viral rebound after analytical treatment interruption (ATI) in individuals treated as early as a few months after seroconversion (91) PD-1-expressing HIV-specific CD8+ T-cells with co-expression of other inhibitory receptors such as Tim-3, CTLA-4, or Lag-3, commonly have the phenotype of exhausted T-cells with impaired effector functions (89, 92). However, the population of PD-1 expressing CD8+ T-cell is heterogeneous, with a hierarchical loss of functional capacity driven by profound epigenetic and transcriptional adaptations. In chronic infections like HIV, persistent TCR signalling leads to accumulation of inhibitory receptors, leading to a phenotype of terminal exhaustion with a progressive decrease in proliferative capacity, IL-2, TNF-α and ultimately in IFN-γ and cytotoxic granule release (93). An intermediate population expresses PD-1, but not other inhibitory receptors, and maintains a large capacity of self-renewal and of generating the larger population of effector and terminally exhausted T-cells (**Figure 2**). Stem-like CD8+ T-cells express the transcription factor T-cell factor 1 (TCF-1) involved in maintaining the IL-2/15Rβ pathway and inhibiting the effects of IFN-I. Increased proliferation capacity is a key feature in adult ECs (77, 94), and PD-1+TCF-1+ virus-specific CD8+ T-cells are increased in viraemic controllers in both adult HIV and SIV infection (78). Their frequency is positively correlated with *in vitro* expansion and memory capacity.

The tolerogenic and non-inflammatory environment observed in infants, especially in PNPs, favours the development of a stem-like CD8+ T-cell population with higher expansion potential not yet explored in HIV infection. We recently observed a correlation between higher frequency of PD-1+ CD8+ T-cells and slower disease progression in a group of 1-year-old infants who undergone ATI after one year on therapy (95) a finding similar to that described in the early resolution of immune activation in non-pathogenic models of SIV infection (96). Moreover, this PD-1 expressing population expressed lower levels of Tim-3 in the slow progressor group, reflecting a non-terminally exhausted phenotype and a possible role for PD-1 in dampening chronic inflammation. Indeed, PNPs in late childhood/adolescence are enriched with a TCF-1+PD-1+ HIV-specific CD8+ T-cells with higher expansion capacity when compared to paediatric progressors and ART-naïve adults (95).

The regulatory effect of PD-1 in controlling CD8+ T-cell activation and exhaustion thus acts as damage control and, like the proposed in the “honeymoon period”, protects the immune system from an unrestrained cycle of inflammation (97) and possibly plays a role in containing disease progression in both in the natural hosts of SIV infection (95, 96) and in PNPs (95). The inhibitory effect of PD-1 contributes to avoiding hyperactivation and terminal differentiation of the CTL population, supporting the generation of a CD8+ T-cell compartment that is non-terminally exhausted but with high proliferative potential and capable of acquiring effector functions. Early treated infants have a marked decrease in the T-cell immune activation compared with infants with delayed ART initiation (16).

The inherently less combative immune system in early life, combined with the opportunity to start ART immediately after birth, creates an ideal scenario that protects the T-cell compartment from terminal exhaustion up to the time when the optimal window of opportunity opens to elicit an antiviral response that is long-lasting and capable of eliminating reactivated cells, and cART can be discontinued.

## Antiviral Immune Responses Important for Remission: Natural Killer Cells

In adult HIV infection, there are particular ‘protective’ (HLA-B*57/58:01/81:01) and ‘disease-susceptible’ (HLA-B*18:01/45:01/58:02) HLA-I molecules that have a significant impact on immune control (45–48). As stated above, these ‘protective’ and ‘disease-susceptible’ HLA-B molecules that affect disease progression in adult HIV infection have a much more modest effect on disease outcome during paediatric HIV infection (32). Less is known about the part played by NK cells in immune control of HIV in early life and the potential for NK cell-based immune interventions to facilitate HIV remission in paediatric infection. In children, NK cells achieve their full effector potential within the first months (98–100) of post-natal life, therefore several years before the HIV-specific T-cell response has become fully effective. The effector potential of NK cells in early life combined with specific genotypes previously linked with HIV disease protection discussed below point to the NK response having a more central role in immune control in early life.

Overall, protection against HIV disease progression and viraemic control in adults has been consistently associated with a KIR-educated program that decreases the amount of NK cell inhibition *via* NKG2A in individuals with HLA-I molecules expressing the HLA-Bw4 motif, with HLA-B alleles expressing Threonine at position -21 (-21T) and with low-expressing HLA-A alleles. KIR3DL1, in particular high-expressing allotypes (KIR3DL1*h), combined with HLA-Bw4-80I is associated with slow HIV disease progression and viral control (99).The combination KIR3DL1*h-HLA-B57 is significantly enriched in adult ECs (101). Moreover, -21T HLA-B molecules offer poor binding for the HLA-E surface expression, decreasing inhibition *via* NGK2A. NKG2A+ NK cells from M/T and T/T individuals living with HIV showed increased cytotoxic and killing capacity compared to those from M/M donors, highly inhibited by the HLA-E interaction (102). Similarly, low expressing HLA-A alleles decrease NK cell inhibition *via* NKG2A by decreasing HLA-E expression, correlating negatively with plasma viral load and positively with CD4+ T-cell count in adult HIV infection (103).

A large cross-sectional paediatric study associated the presence of KIR3DL1-HLA-Bw4 with lower plasma viral load and higher CD4+ T-cell counts in all age bands (104). Our group recently identified an immunogenetic signature of low-HLA-A expression and the presence of HLA-Bw4 expressing HLA-I alleles, favouring KIR-biased education of NK cells, that was associated with slow progression and superior immune control of HIV in ART-naïve children, while ‘protective’ and ‘disease-susceptible’ HLA-B molecules had no impact and clearly contrasted with the adult analysis. It is noteworthy that HLA-B*58:02, the HLA-B molecule with the greatest detrimental impact on immune control in ART-naïve African adults, unusually for a disease-susceptible allele also expresses the Bw4 motif. Bw4 contributes to antiviral NK activity *via* interaction with its cognate receptor KIR3DL1. However, in ART-naïve children, a disease-susceptible effect of HLA-B*58:02 is only evident after 10 years of age, by which time its negative impact on HIV-specific CTL activity is starting to be felt (72). By contrast the disease-susceptible Bw6-expressing alleles HLA-B*45 and HLA-B*18 have a marked detrimental impact during the first 10 years of life (72). These findings are consistent with the evidence that NK cell responses play a more significant role in immune control of HIV infection in early life compared to CTL responses.

In the VISCONTI cohort of adult PTC, the HIV-specific CD8+ T-cell responses were no more potent than those of non-controllers, and the cohort was enriched for disease-susceptible HLA-I alleles, suggesting a mechanism independent of strong CTL activity (10). Subsequent studies in this cohort have shown an immunogenetic signature like the observed in children with better immune control, comprised of disease-susceptible HLA-B molecules in combination with expression of the Bw4 motif, -21T HLA-B alleles and C2 group HLA-C alleles (C2 alleles are KIR2DL1 ligands and promote stronger KIR binding than C1 alleles) (105). This immunogenetic signature implicates a principal role for KIR-educated rather than NKG2A-educated NK cells in achieving functional cure after cART cessation (106).

Paediatric immune control is associated with a less mature NK subset with higher plasticity potential populated by CD56^dim^ NK cells expressing the inhibitory receptor NKG2A and the natural cytotoxicity receptor NKp46 (72). Functionally, these NK cells were highly responsive to cytokine stimulation and missing-self activation. Although not solely explained by immunogenetic, a signature favouring lower expression of HLA-E and a KIR-education program translates phenotypically into a larger NKG2A+ NK cell population (107). NKG2A+ NK cells, particularly from -21T HLA-Bw4 donors, have been linked with higher polyfunctionality and degranulation *via* missing-self assays and higher killing capacity against autologous HIV infected-CD4+ T-cell targets (102, 108). This effect was only observed in HLA-Bw4 donors. Interestingly, preliminary data from an early-ART-treated cohort of infants in Botswana demonstrated an association between CD56^dim^ NK cells expressing another natural cytotoxicity receptor NKp30 and low intact proviral reservoir size within 3 months of birth (16).

Taken together, these data indicate an important role for NK responses in early cART-treated children expressing a favourable immunogenetic signature that reflects KIR-biased education of NK cells. In a recent study of SHIV infected NHP, remission was achieved in a group of ART suppressed monkeys after infusion of PGT121, a broadly neutralising antibody (bnAb), in combination with a TLR-7 agonist, and part of the antiviral effect after ATI was attributed to NK cells (109). An NK cell population with higher plasticity and responsiveness to cytokines renders a higher potential to be exploited in therapeutic interventions, and that can be potentially replicated by damping inflammation with early suppression of infants on ART.

## Impact of Immune Sex Differences and Early Life Innate Immunity

In several studies of *in utero* MTCT, it has been noted that females are 1.5-2-fold more susceptible than males (15, 16, 110–112). A clue to the mechanism underlying this comes from the observation that female fetuses are especially susceptible to MTCT (2–2.5-fold greater than males) when the mother is recently infected (seroconverting or first diagnosed during the pregnancy) whereas no significant sex difference in susceptibility MTCT was present if the mothers were chronically infected (known seropositive prior to the pregnancy). This finding in relation to fetal transmission may be linked to two observations from adult studies: first, in adult-to-adult transmission, the transmitted virus is highly IFN-I-resistant (113–115) but, after some months, the predominant circulating form of virus is IFN-I sensitive (113, 115). Second, plasmacytoid dendritic cells (pDCs), the major producers of IFN-I (116–122), produce more IFN-I in female adults when exposed to HIV than in male adults (123). We hypothesised from these data that female fetuses, like their adult counterparts, have stronger IFN-I responses than male fetuses. This would lead to higher levels of immune activation and of CCR5 expression on CD4+ T-cells *in utero*, thereby increasing susceptibility to HIV infection in female fetuses, and, in particular, to the IFN-I-resistant viruses that would be the major circulating form of virus following recent maternal infection. By contrast, male fetuses, having weaker IFN-I responses, are less prone to immune activation following exposure to HIV, and therefore less susceptible to MTCT of HIV irrespective of IFN-I sensitivity.

In studies of cord blood from sex-discordant twins born to HIV-uninfected mothers (124), cord blood CD4+ T-cells were indeed more activated in females than males and also were more susceptible to *in vitro* infection by HIV. This *in vitro* susceptibility was correlated with the levels of immune activation and CCR5 expression on CD4+ T-cells. Analysis of the viruses that were transmitted *in vivo* to male and female fetuses, respectively, showed that female fetuses were indeed more susceptible to IFN-I-resistant viruses compared to males. Male fetuses, on the other hand, were more susceptible to IFN-I-sensitive viruses. As mentioned above, the viruses transmitted *via* MTCT tended to have a lower replicative capacity than those circulating in the mother, but whereas viruses of very low replication capacity could infect female fetuses, male fetuses typically were infected only by viruses of relatively high replicative capacity.

**Figure 3:**
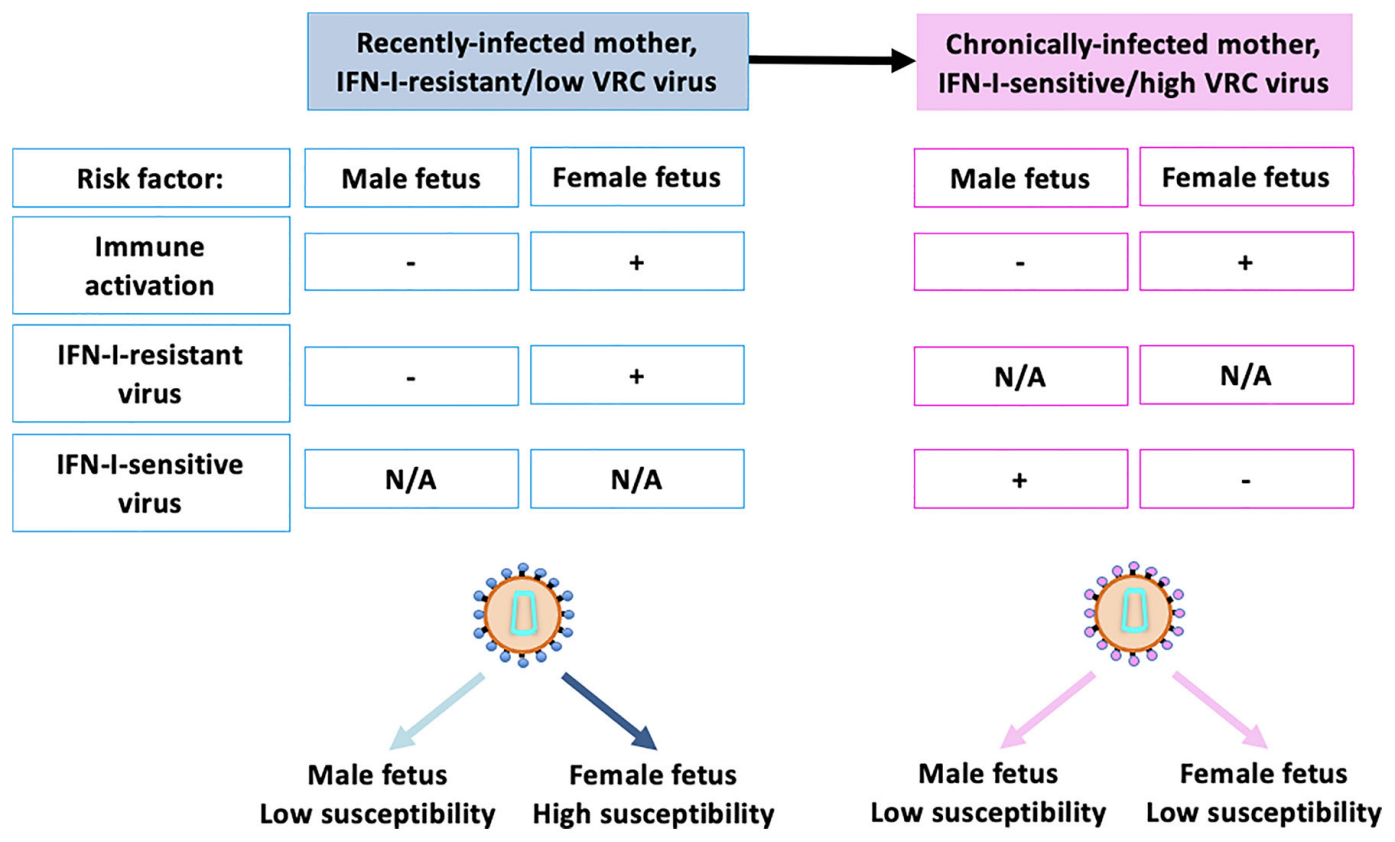
Key risk factors contributing to sex differences in susceptibility to *in utero* infection. Recently-infected mothers typically carry highly IFN-I-resistant virus which tends have lower replicative capacity than IFN-I-sensitive virus. Female fetuses have higher levels of immune activation, even in the absence of exposure to HIV, and are also especially susceptible to infection to IFN-I-sensitive viruses. Male fetuses are not as susceptible to IFN-I-resistant viruses, for reasons incompletely understood, although this may be related to the fact that IFN-I-resistant viruses tend to have low replicative capacity. These factors together result in female fetuses being 2–3x more susceptible to *in utero* MTCT than male fetuses in the setting of recent maternal infection. Chronically infected mothers typically harbour IFN-I-sensitive viruses, to which male fetuses are somewhat more susceptible. Thus, taking into account the greater immune activation of female fetuses, male and female fetuses have similar susceptibility in the setting of chronic maternal infection. N/A, Not Applicable.

These data suggest that female fetuses are more susceptible to MTCT in part because their immune system is more readily activated *in utero*, as a consequence of greater IFN-I production by innate immune cells such as pDCs; and, as a result, they are more susceptible to infection *via* the IFN-I-resistant viruses that are prevalent following recent maternal infection. The replicative capacity of the virus appears to a less important factor than IFN-I resistance in MTCT to female fetuses. For male fetuses with relatively low IFN-I production in response to HIV exposure, however, their immune systems do not activate so readily, and they are less susceptible to infection by HIV, irrespective of IFN-I sensitivity. However, males are more susceptible to viruses with high replicative capacity, and these tend to be IFN-I-sensitive (**Figure 3**).

Further work is needed to determine the extent to which these tentative conclusions are justified. However, it is apparent that immune sex differences are present *in utero* that significantly increase the susceptibility of female fetuses to *in utero* HIV infection and that there is sex-specific selection of viruses transmitted *via* MTCT on the basis of viral IFN-I sensitivity. This implicates the IFN-I response *in utero* as playing a critical part in the defense against *in utero* HIV infection, especially in female fetuses, because of sex-specific adaptation of the virus to the innate immune system shared by mother and daughter.

What impact might these immune sex differences *in utero* have on subsequent HIV cure potential? In adult studies it is noteworthy that, while IFN-I-resistant viruses are transmitted, the viruses that rebound following cART interruption are even more IFN-I-resistant (115). Thus, there may be an advantage in male fetuses being infected by IFN-I-sensitive virus, in that local control of productive viral replication in lymphoid tissues by innate immunity may delay viral rebound. Preliminary studies of early-cART-treated children in a setting of poor cART adherence indicated that viral rebound does indeed happen more rapidly among females, for reasons not explained by gender differences in cART adherence (124).

In females, however, there are some potential advantages in terms of the likelihood of achieving cure. First, female fetuses are infected with lower replication capacity virus than the males. As mentioned above, adult studies have shown this correlates with low levels of immune activation and low reservoir size (36). Second, the higher levels of IFN-I response we have inferred are present in females from fetal life onwards may be of benefit in delaying viral rebound if cART interruption can be delayed some years into childhood. In the SPARTAC all-female adult cohort, CD4+ T-cell expression of IFN-I stimulated genes were associated with a delayed time to viral rebound following cART interruption (125). Certainly, in cART-naïve children above 2 years of age, but not before (126), immune control of HIV is markedly more efficient among females than males.

In summary, the IFN-I response is a double-edged sword, and the effects of a stronger IFN-I response can be highly unpredictable. For example, it is now well-established that immune sex differences typically result, in female adults and children, in superior disease outcomes from infections and stronger antibody responses to vaccines; but, also in females, in more immunopathology, autoimmune disease, and adverse events from vaccines (127, 128). During the early stages of adult HIV infection, immune control is superior among females (129), who are 5 times more likely to achieve ‘elite control’, than in males (130). But in chronic HIV infection, increased immune activation of the female immune response is in fact a disadvantage, as it essentially ‘fuels the fire’ of HIV replication (131), reflected in more rapid disease CD4+ decline for a given viral load in females compared to males. Susceptibility to HIV infection amongst adolescents is skewed 3:1 towards females (132). Whilst cultural and socio-economic factors clearly play an important role, it is possible from the immune sex differences described in fetal life, which also results in a 2-fold increased risk of MTCT of hepatitis C virus infection (133, 134) as well as of HIV infection, that biological factors unrelated to behavioural and anatomical sex differences may contribute.

## bnAb Interventions in Paediatric Infection

Broadly neutralising antibodies (bnAbs) have been developed as antiviral agents and when administered passively have certain advantages over cART, such as lower toxicity and potential for less frequent administration (135), overcoming adherence issues. In non-human primates, the administration of bnAbs have facilitated the attainment of true or functional cure following SHIV infection either when given early following infection in the absence of ART (136) or when used in combination with a TLR7 agonist during ART (136). These data suggest that the administration of bnAbs in the presence of innate immune stimulation could reduce viral reservoir size (109) and that virus-specific CTL could also play a central part in maintaining functional cure. Such findings have led to studies investigating whether viral rebound following ATI could be delayed or prevented by passive bnAb infusions (137, 138). These clinical studies have shown that bnAb delays viral rebound after ATI, highlighting their potential role in achieving long term remission or cure. The mechanisms by which the administration of bnAbs delays viral rebound include bnAb-mediated killing of HIV-infected cells through antibody-dependent cellular cytotoxicity (ADCC), by direct antiviral NK activity and by CTL responses boosted *via* vaccinal effects (136).

ADCC activity occurs when the Fab region of antibodies binds to the Env protein on the surface of HIV-infected cells. Fc receptors on immune cells, such as FcgRIIIa or CD16 on NK cells (139) are then able to bind to the Fc portion of the bound antibody. Once the antibody-mediated bridge between the infected cell and the immune cell is established, the immune cell kills the infected cell *via* ADCC. In the context of HIV infection, passively acquired ADCC activity has been associated with improved survival in HIV-infected children (140–142). The maternal HIV-specific antibodies, responsible for the passively acquired ADCC activity, enter circulation of the infant *via* the placenta and remain in circulation for months after birth (140, 143–145). Although ADCC activity is classically thought to be mediated by NK cells, a growing body of literature supports a potential role for monocytes also in promoting ADCC and facilitating protective effects against HIV (146–150). The ability of the infant innate immune response to mediate ADCC therefore does plays a part in the control of viraemia and must therefore be taken into account when administering therapeutic interventions, such as passive bnAb immunotherapy. With this in mind, it is relevant to note that, in African populations, 80–90% of infants are CMV-infected by 12 months (151, 152). CMV infection profoundly expands the memory-like NK cells up to 70% of total NKs (153). These have enhanced cytotoxic and ADCC functionality (154, 155) and therefore the potential to amplify the antiviral impact of bnAbs. Infants co-infected with CMV and HIV may therefore benefit from the increased ADCC functionality which could potentially influence the impact of bnAbs in mediating immune control of HIV.

In cohorts where infected children are treated with ART within minutes birth, the administration of bnAbs at ATI during the age of 5 to 10 years old could provide a unique opportunity to facilitate the elimination or reduction of viral reservoirs to achieve true or functional cure. The optimal timing of the intervention is unknown but the ‘honeymoon period’ of infectious disease mentioned above, is attractive in that the HIV-specific CD8+ T-cell response begins to play a prominent role in control of viraemia at this time. This CTL response could be boosted in a vaccinal manner by the administrated bnAbs, to operate alongside ADCC-mediated killing by NK cells. Further benefits to timing a bnAb intervention and analytical treatment interruption prior to adolescence are that the risk of HIV transmission resulting from viral rebound is low and cART adherence in children prior to adolescence is relatively high.

## Impact of the cART Era on Paediatric Cure Potential

As mentioned above, cART has transformed outcomes in adults and children living with HIV, as well as decreasing transmission risk in serodiscordant couples to zero when plasma viral load is suppressed by cART to undetectable in the seropositive partner (156). In the pre-cART era, MTCT was strongly associated with maternal viral load (157) and cART in pregnancy and post-natally has reduced rates of *in utero* and intra-partum MTCT from approximately 25% to <2% (158, 159), and rates of post-partum (breast-feeding) transmission from approximately 15% to ~2% (160). However, since cART has become available to mothers testing seropositive in pregnancy, there have also been changes in the genetic factors associated with MTCT, as described below. In turn, these changes in the genetic make-up of the children who currently become infected *via* MTCT, and towards whom efforts to achieve cure/remission are now directed, can be expected to have an impact on cure potential in children and on the approaches that may be successful in attaining it.

In the pre-cART era, MTCT was strongly related to maternal viraemia and therefore HLA class I molecules associated with high viral load (‘unfavourable alleles’), such as HLA-B*18/45:01/58:02 (161) might be expected to be expressed at higher frequency in transmitter mothers than non-transmitter mothers; and HLA class I molecules associated with low viral load, such as HLA-B*27/57/58:01/81:01 (‘favourable alleles’) (161) to be expressed at lower frequency in transmitter mothers. This indeed proves to be the case: in the pre-cART era, the frequency of unfavourable alleles were 44% and 47% in transmitter mothers and their children, respectively, compared with 26.5% in non-transmitter mothers (162) and the frequency of favourable HLA-I was 17% and 17.5% in transmitter mothers and their children, respectively, versus 31% in non-transmitter mothers (156).

In the current cART era, by contrast, the mothers who transmit are those who have not been able to access cART, either because they seroconverted and/or were diagnosed late in pregnancy (in 70–75% of cases in our study (15). In contradistinction to the pre-cART era, therefore, the reason these mothers have transmitted would not be related to the HLA-I molecules expressed. Indeed, the ‘chronic’ group of mothers (that is, those known to have seroconverted prior to the pregnancy) include an increasing number (9% in our cohort (15),) of mothers who themselves were infected *via* MTCT. The fact that they have been able to survive 20 years (the median age, in our cohort) with HIV, having lived through at least the first 5 years of their lives without cART, during which the 2-year mortality in ART-naïve children was greater than 50% (37) means that they likely carry HLA-I and other genes that *protect* against HIV disease progression (163). Indeed, in the cohort of transmitter mother-child pairs we have followed in the cART era, the frequency of unfavourable and favourable HLA-I is, respectively, 32%-33% and 27%. These are not significantly different from the frequencies of unfavourable and favourable HLA-I in people living with HIV in the same location in KwaZulu-Natal, South Africa (29% and 28.5%, respectively).

Similar findings are to be expected in relation to the favourable, low-expressing HLA-A molecules associated with low viraemia, such as HLA-A*03/32/33/74 and the unfavourable, high-expressing HLA-A molecules such as A*01/24 (164), *via* their impact on NK responses. However, as explained above, whilst differences in HLA-A molecules have an impact on viral load in adult infection *via* the effect on antiviral NK responses, this effect in adults is less dramatic than that mediated by differences in the HLA-B molecules expressed that impact on effectiveness of CTL-mediated immune control of viraemia (161). Thus, the benefits of the cART era to the antiviral immune responses that can be raised by infected children against HIV may be manifested more in enhanced HIV-specific CTL responses than in improved NK activity. These changes in the paediatric HIV epidemic may have implications for the approaches most likely to be successful in achieving cure/remission in paediatric infection, with therapeutic T-cell vaccines being more likely to have an impact in the setting of favourable HLA-I expression (165).

## Conclusions: Implications for Strategies to Achieve Cure/Remission in Paediatric HIV

**Figure 4:**
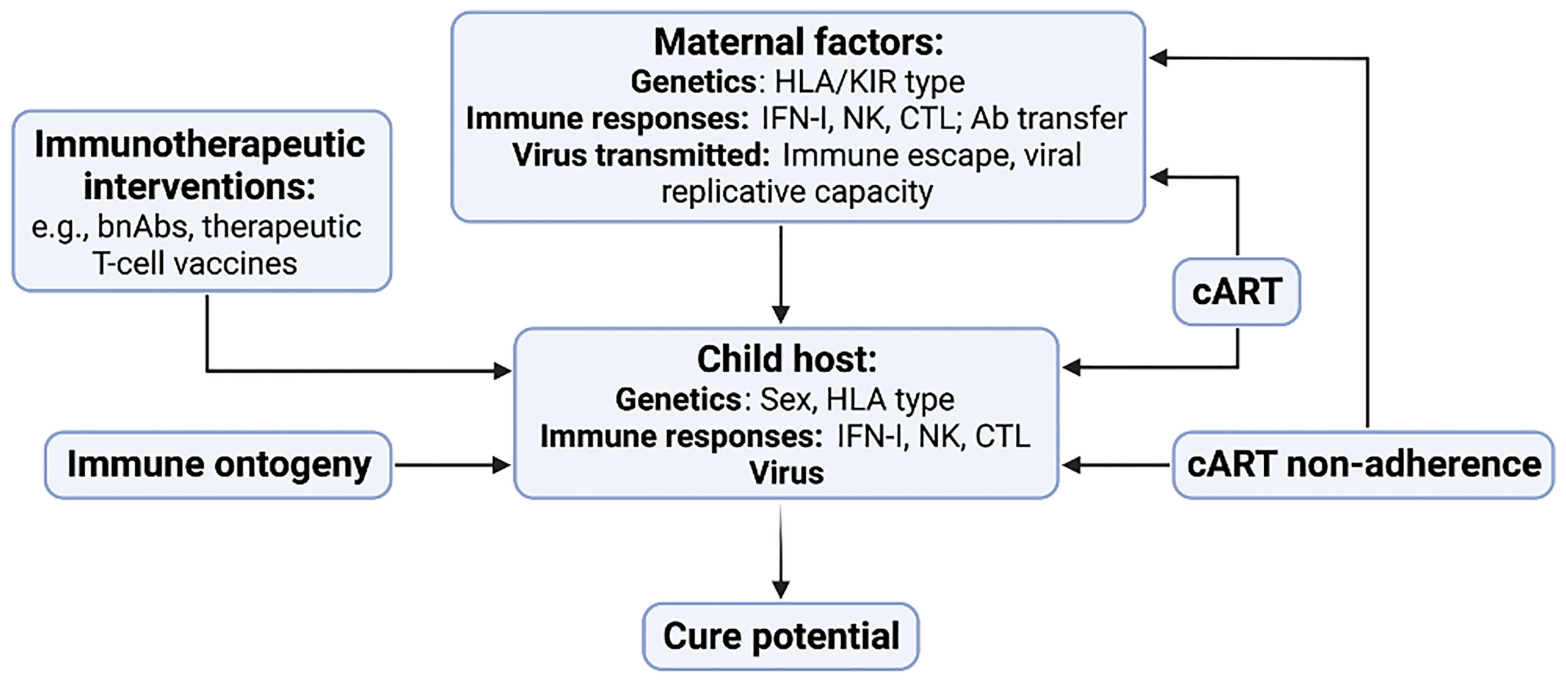
Key factors contributing to HIV cure potential in children with HIV. Maternal factors include host genetics, immune responses and the virus that is transmitted to the child. Factors in the child include genetic factors such as sex and HLA type, the innate and adaptive immune response, and the nature of the virus that was transmitted. cART has strong influences on the immunogenetics of transmitting mother-child pairs in addition to its early initiation following MTCT resulting in a small and low-diversity reservoir in many children. Changes in the child’s immune response resulting from immune ontogeny, in particular the increasing antiviral efficacy of the HIV-specific CTL response with increasing age, will affect the impact of immunotherapeutic interventions such as bnAb therapy and T-cell vaccines.

This review highlights the key roles of several interdependent factors in influencing HIV cure potential in children (**Figure 4**). At the centre is the child host, the virus that has been transmitted, and the antiviral immune responses available at different ages with which to eradicate it. But the influence of the mother is also central, since she shapes the immunogenetics in the child as well as the virus that is transmitted. The timing of maternal infection in relation to MTCT, and the maternal immune response to the virus, strongly influence the virus that is transmitted, and, in particular, the degree of viral adaptation to maternal immunity and therefore also to the child’s immune responses that are shared with the mother. A further unique aspect of paediatric versus adult infection is the transplacental transfer of maternal antibody, which may affect the sensitivity of transmitted virus to future passive bnAb immunisation treatments in the children.

cART has two critical effects, distinct from that of preventing transmission and improving disease outcome: first, when administered to mothers during pregnancy, it results in children infected *in utero* receiving cART prior to birth, consequently in many cases with their having very small and low-diversity viral reservoirs at birth; and, second, cART administered to mothers prior to or early in pregnancy has interrupted the process of natural selection that previously had resulted in high expression of unfavourable HLA-I in transmitter mother-child pairs. Finally, despite the existence of a handful of anecdotal cases of cure/remission in children, it is evident that early cART alone will only in exceptional cases be sufficient to achieve it, and it is clear that some immunotherapeutic intervention in addition to early cART initiation will be needed, from the range of options including passive bnAb immunisation and the use of therapeutic vaccines to boost antiviral T-cell immunity. The optimal timing of such interventions is unknown but will be affected by the specific immune responses that are being targeted, as well as by practical considerations such as cART adherence. For example, HIV-specific CTL responses become more efficacious with increasing age but cART adherence in adolescence is notoriously challenging, even after years of faultless adherence (23). The window of opportunity may be relatively small, therefore, for interventions to achieve cure in early-cART treated children. This, after all, is a major reason why efforts to achieve cure need to be pursued: it is not realistic to expect children to remain on cART from birth throughout the normal lifespan.

## Author Contributions

VV, NH, EA, GC and PG all contributed equally to the review process - reviewing, writing and figure design. PG is the senior author. All authors contributed to the article and approved the submitted version.

## Funding

This work was supported by the Wellcome Trust (PG WTIA Grant WT104748MA), the National Institutes of Health (PG RO1-AI133673, 1UM1AI164566-01) and by a grant to PG through the EPIICAL Project (Early-treated Perinatally HIV-infected Individuals: Improving Children’s Actual Life with Novel Immunotherapeutic Strategies). The EPIICAL Project is funded through an independent grant by ViiV Healthcare UK.

## Conflict of Interest

The authors declare that the research was conducted in the absence of any commercial or financial relationships that could be construed as a potential conflict of interest.

## Publisher’s Note

All claims expressed in this article are solely those of the authors and do not necessarily represent those of their affiliated organizations, or those of the publisher, the editors and the reviewers. Any product that may be evaluated in this article, or claim that may be made by its manufacturer, is not guaranteed or endorsed by the publisher.
